# Synthesis and Characterization of FITC Labelled Ruthenium Dendrimer as a Prospective Anticancer Drug

**DOI:** 10.3390/biom9090411

**Published:** 2019-08-25

**Authors:** Sylwia Michlewska, Małgorzata Kubczak, Marta Maroto-Díaz, Natalia Sanz del Olmo, Paula Ortega, Dzmitry Shcharbin, Rafael Gomez Ramirez, Francisco Javier de la Mata, Maksim Ionov, Maria Bryszewska

**Affiliations:** 1 Laboratory of Microscopic Imaging and Specialized Biological Techniques, Faculty of Biology and Environmental Protection, University of Lodz, Banacha12/16, 90-237 Lodz, Poland; 2 Department of General Biophysics, Faculty of Biology and Environmental Protection, University of Lodz, Pomorska 141/143, 90-236 Lodz, Poland; 3 Networking Research Center on Bioengineering, Biomaterials & Nanomedicine (CIBER-BBN), Monforte de Lemos 3-5, Pabellon´ 11, Planta, 028029 Madrid, Spain; 4 Departamento Química Orgánica y Química Inorganica, Universidad de Alcalá, Instituto de Investigación Química “Andrés M. del Río” (IQAR), UAH, 28871 Alcalá de Henares, Spain; 5 Institute of Biophysics and Cell Engineering of NASB, Akademicheskaja 27, 220072 Minsk, Belarus; 6 Instituto Ramon y Cajal de Investigacion Sanitaria, IRYCIS, Colmenar Viejo Road, Km 9, 100, 28034 Madrid, Spain

**Keywords:** ruthenium dendrimer, FITC labelled dendrimer, anticancer therapeutic agent, controlled polymer carrier

## Abstract

Metallodendrimers—dendrimers with included metals—are widely investigated as biocompatible equivalents to metal nanoparticles. Applications can be expected in the fields of catalysis, as chemical sensors in molecular recognition and as anticancer drugs. Metallodendrimers can also mimic certain biomolecules, for example, haemoprotein in the case of using a dendrimer with a porphyrin core. In previous papers, we showed the promising anticancer effects of carbosilane ruthenium dendrimers. The present paper is devoted to studying biocompatibility and the cytotoxic effect on normal and cancer cells of carbosilane ruthenium dendrimers labelled with fluorescent probe fluorescein isothiocyanate (FITC). The addition of fluorescent probe allowed tracking the metallodendrimer in both normal and cancer cells. It was found that carbosilane ruthenium dendrimer labelled with FITC in concentration up to 10 µmol/L was more cytotoxic for cancer cells than for normal cells. Thus, FITC labelled carbosilane ruthenium dendrimer is a good candidate for diagnostic imaging and studying anticancer effects of metallodendrimers in cancer therapy.

## 1. Introduction

Cancers are the second main cause of mortality in the world after cardiovascular diseases. The wide heterogeneity of cancers and their metastasising pose huge challenges for discovering new methods of treatment [1]. Despite the development of many anticancer agents, high mortality often results from the poor, non-selective delivery systems unable to target only cancer cells. Metal compounds, for example cisplatin, are well known anticancer agents due to their mechanism of action [2,3]. Unfortunately, they are highly cytotoxic, not only for tumour tissues, but for the whole body. Cisplatin is widely used in cancer therapy, but its anticancer activity is limited by its adverse side effects (anaemia, diarrhoea, nephrotoxicity and neurotoxicity). Many cancers have become resistant to treatment with this metal [2].

Now, novel metal compounds (zinc, copper, and ruthenium) are under investigation as potential anticancer drugs [2,3,4]. It was demonstrated that ruthenium exhibited anti-tumour activity with limited side effects towards normal cells [5]. Thus, ruthenium-based drugs may be an alternative to more toxic platinum agents. Ruthenium can have II, III or IV oxidation states; the oxidation state III does not damage cells. Due to the low pH and high glutathione level characteristic of cancer cells, ruthenium can be reduced to II oxidative state, which is highly toxic towards cells [5]. The detailed analysis of metallodrugs has shown that complexes containing these metal ions are already in clinical use or have advanced to clinical trials as anticancer agents. The “multitargeted” complexes described herein not only target DNA but also contain vectors to enable them to target cancer cells selectively and/or moieties that target enzymes, peptides, and intracellular proteins [6,7]. The anticancer activities of ruthenium include inhibition of DNA and RNA synthesis, which leads to apoptosis. However, the biggest impediment for use of ruthenium compounds is their poor solubility in water [8]. Therefore, creating ruthenium compounds that are soluble in water and can transfect cancer cells is important [9]. Currently, the attention of scientists is focused on controlled delivery of cancer therapies or on targeted diagnostic imaging [10,11]. The use of nanotechnology seems to be very promising. Targeted use of a drug, with the possibility of imaging the process of its application, will increase therapeutic efficacy and further minimise side effects [12]. For many years, nanoparticles, especially dendrimers, have been under investigation as systems for delivery of drugs to cancer cells. Dendrimers are very convenient subjects to study [13]: they are synthesised in a controlled manner; their molecular weight, surface charge and size are well defined; and they consist of a core and polymer branches. The ends of polymer branches can be modified in different ways. Due to these features, they are promising agents to be used in many scientific fields, especially in gene and drug delivery systems [14]. Their low immunogenic activity and cytotoxicity make them attractive carriers for poorly-soluble drugs or nucleic acids. Gene therapy seems a very promising method to overcome many cancers but finding the most effective gene delivery system is still a challenge. The hydrophobic nature and stability of carbosilane ruthenium dendrimers may facilitate their interaction with biological membranes. Additionally, it is known that they can create complexes with nucleic acids [14,15,16]. Big progress in metallodendrimers was achieved at the University of Cape Town [17]. They developed a wide range of Ru-based metallodendrimers and studied their effects in vitro against A2780 and A2780cisR human ovarian cancer lines, the SISO human cervix cancer line, the LCLC-103H human lung cancer line, and the 5637 human bladder cancer line. Nine of the twelve compounds slowed the growth of the ovarian cancer cell lines by more than 50% at equi-iron concentrations of 5 μmol/L [17]. Except dendrimers, other nanoformulations are used to improve the delivery of ruthenium into cancer cells. Heffeter et al. [18] presented a new nanoparticle formulation based on polymer-based micelles loaded with the anticancer lead ruthenium compound KP1019. Nanoprepared KP1019 was characterised by enhanced stability in aqueous solutions. Moreover, the nanoparticle formulation facilitated cellular accumulation of KP1019 (determined by Inductively coupled plasma mass spectrometry (ICP-MS) measurements) resulting in significantly lowered IC_50_ values [18]. The KP1019 poly(lactic acid) nanoparticles of KP1019 with two different surfactants (Pluronic F68 and Tween 80) were prepared [19] and cytotoxicity measurements comparing different aged Tween 80 nanoparticles revealed that the colour change from brown to green was associated with an up to 20 fold increased activity compared to “free” KP1019 [19]. Hollow mesoporous ruthenium nanoparticles conjugating bispecific antibody for targeted anti-colorectal cancer response of combination therapy were proposed by Xu et al. [20]. Systematic in vivo studies clearly demonstrated the high tumour targeting and anticancer activity in heterotopic colorectal tumour model via combined photothermal and immune therapy [20]. As for imaging, Dou et al. [21] proposed a ruthenium-complex-functionalized two-photon-excited red fluorescence silicon nanoparticle composite based on fluorescence resonance energy transfer under mild experimental conditions. Fluorescence imaging tests in zebrafish and nude mice indicated that such nanoparticle could act as a new kind of fluorescence probe for fluorescence imaging in vivo. The present paper is devoted to studying biocompatibility and cytotoxic effect on normal and cancer cells of carbosilane ruthenium dendrimer labelled with fluorescent probe fluorescein isothiocyanate (FITC). The addition of a fluorescent probe allows tracking the metallodendrimer in normal and cancer cells.

## 2. Materials and Methods

### 2.1. Ruthenium-Terminated Carbosilane Dendrimer with FITC (CRD13-FITC)

The new heterofunctional ruthenium-terminated carbosilane dendrimer with FITC (CRD13-FITC), G1-{[[NCPh(o-N)Ru(η6-p-cymene)Cl]Cl]_3_[FITC]} (**3**), was prepared as described below and characterized through Nuclear Magnetic Resonance, Elemental Analysis, UV-Vis and IR spectroscopy (see the Supplementary Materials for structural characterization).

G1-[(NCPh(o-N))_3_NH_2_] (**1**). Carbosilane dendrimer G1-[NH_2_]_4_ (313.1 mg 0.47 mmol) was dissolved in THF, and 2-pyridinecarboxaldehyde (152.1 mg, 1.42 mmol) was added. The mixture was stirred in an inert atmosphere at room temperature in anhydrous MgSO_4_ for 12 h. The solution was filtered and the solvent evaporated to isolate dendrimer **1** as an orange oil (354.1 mg, 80%). This reaction was controlled by ^1^H-NMR. The corresponding aldehyde was added in portions. By using NMR, the percentage of amino groups that had reacted was calculated; thus, the reaction could be stopped when there were 25% unreacted amino groups. Finally, we carried out the experiment of elemental analysis to corroborate the purity of the final product.

G1-[(NCPh(o-N)Ru(η6-p-cymene)Cl_2_)_3_NH_2_] (**2**). Compound **1** (96.2 mg, 0.104 mmol) was dissolved in EtOH and added dropwise at 0 °C to a solution of [Ru(η6-p-cymene)Cl_2_]_2_ (95.5 mg, 0.156 mmol) in EtOH. The mixture was stirred at r.t. for 12 h. Subsequently, the solvent was evaporated to isolate compound **2** as a water-soluble red solid (115.1 mg, 60%).

G1-{[[NCPh(o-N)Ru(η6-p-cymene)Cl]Cl]_3_[FITC]} (**3**). Compound **2** (90 mg, 0.049 mmol) was dissolved in EtOH and FITC (1.0 eq.) was added. The mixture was stirred under dark and inert atmosphere at 55 °C for 12 h. Subsequently, the solvent was evaporated and the product was washed with water and dialyzed with a membrane with molecular weight cut-off 100–500 Da for 72 h. Compound **3,** referred to here as CDR13-FITC, was isolated as a brown solid (65.8 mg, 60%).

### 2.2. Zeta Potential

Zeta potential of CRD13-FITC was measured using a Photon Correlation spectrometer Zetasizer Nano ZS Malvern Instruments (Malvern Instruments Limited, Worcestershire, UK). Measurement of 20 μmol/L dendrimer was made in distilled water at 25 °C. The values were calculated from the Helmholtz-Smoluchowski equation. From 9 to 12 measurements were collected and averaged for each sample.

### 2.3. Measurement of Particle Size

The size of CRD13-FITC dendrimer at 20 μmol/L was measured using Malvern Zetasizer Nano-ZS (Malvern Instruments Limited, Worcestershire, UK) spectrometer by dynamic light-scattering technique. The wavelength was set to 633 nm, the detection angle to 90°, and the refraction factor to 1.33. All samples were prepared in distilled water. Nanoparticle size was measured from the average of 7 × 3 cycles at 25 °C. The data were analysed using Malvern software.

### 2.4. Transmission Electron Microscopy

To examine dendrimer morphology, TEM was used. Ten microliters of dendrimer solution at 1 mmol/L were placed on 200-mesh carbon-coated copper grids. The sample was stained with saturated uranyl acetate for 20 min, washed in demineralised water and dried at room temperature. Images were obtained using a JEOL1010 transmission electron microscope (JEOL, Tokyo, Japan).

### 2.5. Haemotoxicity

Blood obtained from healthy donors was purchased from Central Blood Bank, Lodz, Poland. It was centrifuged and washed twice with PBS at pH 7.4. Erythrocytes were used immediately after isolation. From 0.5 to 50 μmol/L of CRD13 FITC dendrimer was added to the red blood samples (haematocrit 2%) and incubated for 24 h at 37 °C. Haemolysis percentage was calculated as follows:

H(%) = (A_pb_ 540 nm/A_water_ 540 nm) × 100%. (1)

H(%) is the percentage of haemolysis, A_pb_ 540 nm is the absorbance of the erythrocytes incubated with CRD13-FITC, and A_water_ 540 nm is the absorbance of erythrocytes incubated with water (100% haemolysis, positive control).

### 2.6. Fluorescence Anisotropy

Human erythrocyte membranes were used to analyse changes of fluorescence anisotropy. Red blood cells were haemolysed in 30 mM Na-phosphate buffer at pH 7.4 and at 4 °C before being centrifuged (15 min, 15,000× *g* at 4 °C). Membranes were separated from haemoglobin and washed several times with Na-phosphate buffer diluted in water. Protein concentration was estimated by the Lowry method [22]. The erythrocyte membranes were frozen and used within two weeks.

Fluorescence anisotropy of two fluorescent markers, 1,6-diphenyl-1,3,5-hexatriene (DPH) and 1-(4-(trimethyloamino)phenyl)-6-phenylhexa-1,3,5-triene (TMA-DPH), was measured in rising concentrations of CRD13-FITC.

LS-50B (Perkin Elmer, UK) spectrofluorimeter was used to monitor the fluidity of membranes. Excitation and emission wavelengths were 348 nm and 426 nm for DPH and 358 nm and 428 nm for TMA-DPH, respectively. The slit width of the excitation monochromator was 2.5 nm and that for emission monochromator was 6 nm for all samples. The measurements were performed at 37 °C. Membranes were dissolved in Na-phosphate buffer, pH 7.4. the fluorescent probes were added at 25 μg proteins/mL. The concentration of both fluorescent probes was 1 µmol/L. After 10 min incubation, fluorescence anisotropy was measured. CRD13-FITC was dissolved in water and added to each sample to reach the appropriate concentration. Fluorescence anisotropy was calculated using Perkin Elmer software from Jablonski’s equation:

r = (I_VV_ − GI_VH_)/(I_VV_ + GI_VH_) (2)

where r is fluorescence anisotropy, and I_VV_ and I_VH_ are the vertical and horizontal fluorescence intensities, respectively, to the vertical polarisation of the excitation light beam. G = I_VH_/I_VV_ (grating correction factor) corrects the polarisation effects of the monochromator.

### 2.7. Cells

To estimate the cytotoxicity and cellular uptake of CRD13-FITC normal cell line (PBMC) and two cancer cell lines (1301-suspension, human T cell leukaemia and HL-60-suspension, promyelocytic human leukaemia) were used. PBMC was isolated from blood samples by centrifugation in a Histopaque 1077 gradient (1500 rpm, 15 min. at 24 °C) and washed twice in PBS at pH 7.4. The cells were resuspended in RPMI 1640 medium with heat-inactivated 10% FBS and antibiotics (1%) PBMC, 1301 and HL-60 cells were maintained in plastic tissue culture flasks (Falcon) kept at 37 °C in a humidified atmosphere of 5% CO_2_/95% air.

### 2.8. Cytotoxicity

The cytotoxicity of CRD13-FITC dendrimer was estimated using Alamar Blue assay. After 24 and 72 h treatment, viability was calculated from the equation:

% viability = (A − A_0_)/(A_c_ − A_0_) × 100% (3)

where A is the absorbance of sample, A_c_ is the absorbance of control (non-treated cells), and A_0_ is the absorbance of 10% DMSO treated cells (100% cell death, positive control).

### 2.9. Cellular Uptake

CRD13-FITC dendrimers at 2.5 and 5 µmol/L were used to examine cellular uptake by HL-60 and 1301 cells. The cells were seeded 2 × 10 cells/mL in 1 mL RPMI (10% FBS, 100 U/mL penicillin, and 0.1 mg/mL streptomycin) on a 24-well plate (2,000,000 cells/well) and incubated at 37 °C in a humidified atmosphere with 5% CO_2_ in air. After 24 and 72 h incubation, the cells were washed with PBS (1500 rpm, 5 min) and transferred to cytometry tubes. Immediately before measurement, 0.4% Trypan Blue was added to the samples (1:1) to exclude dead cells and cells with attached dendrimers on their surface. The samples were measured in a Becton Dickinson LSRII flow cytometere (BD, Franklin Lakes, New Jersey, USA), with acquisition of 10,000 events using 488 nm excitation wavelength. Emission signal was registered at 530 nm ± 30 nm wavelength. The data were analysed using FlowJo 10 software (BD, Franklin Lakes, New Jersey, USA).

For confocal microscopy, 1301 and HL-60 cells after 24 h incubation with CRD13-FITC dendrimer at 2.5 and 5 μmol/L were stained with Dapi (Thermo Fisher Scientific, Waltham, USA) and Texas Red-X Phalloidin (Thermo Fisher Scientific, Waltham, USA). Images were taken with a Leica TCS SP8 microscope (Wetzlar, Germany) at different wavelengths (405, 495 and 565 nm). Leica software (Wetzlar, Germany) was used to analyse the data.

### 2.10. Statistical Analysis

For haemotoxicity, results were obtained from a minimum of three independent repeats and are presented as mean ± SD. For the Alamar Blue test, the results are shown as mean ± SD. Statistical analyses were performed using Student’s test, with significance set at *p* < 0.05.

## 3. Results

### 3.1. Synthesis of Ruthenium-Terminated Carbosilane Dendrimer with FITC (CRD-FITC)

**Figure 1:**
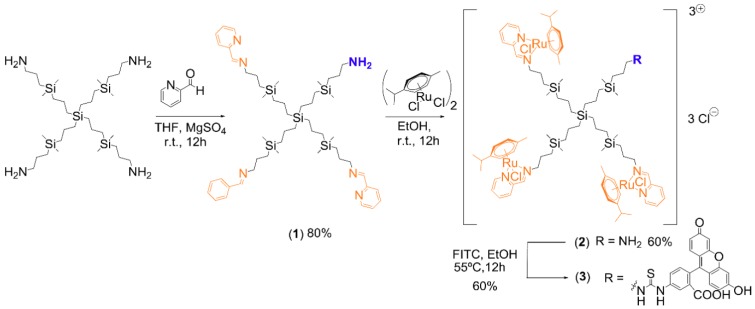
Synthesis of ruthenium-terminated carbosilane dendrimer with FITC (CRD13-FITC).

**Figure 2:**
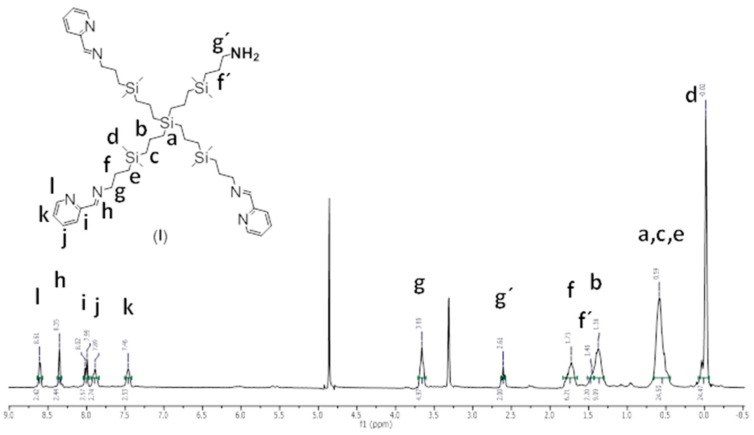
^1^H-NMR spectrum of compound **1** in CD_3_OD.

**Figure 3:**
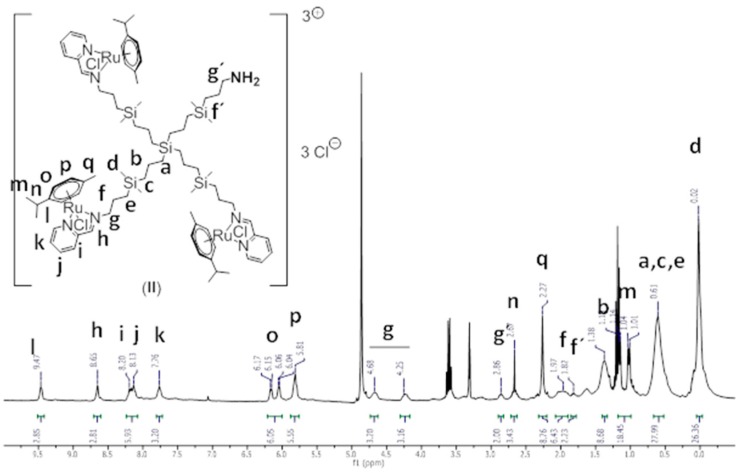
^1^H-NMR spectrum of compound **2** in CD_3_OD.

Synthesis and structural characterisation of CRD13-FITC is presented in detail in Figure 1, in the Methods Section 2. and the Supplementary Materials. In brief, a statistical hetero-functionalisation of carbosilane dendrimer G1-[NH2]_4_ generated dendrimers G1-[(NCPh(o-N))_3_NH_2_] (1), with a distribution of iminopyridine groups and amino moieties in a 3:1 ratio. The metal complexation to the iminopyridine moieties proceeded with a good yield, and this was confirmed by the displacement of the proton signal closest to the metal centre in the ^1^H-NMR spectra (Figure 2 and Figure 3). Metallodendrimer G1-[(NCPh(o-N)Ru(η6-p-cymene)Cl_2_)_3_NH_2_] (2) was isolated as a water-soluble red solid, wherein the presence of a free amino group was confirmed by NMR and a Kaiser test. In the final step, FITC reacted with compound **2** to generate an isothiocyanate bond through the free amino group. FITC-labelled hetero-functional CRD13 was characterised through NMR, DOSY and UV-Vis experiments, confirming the binding of fluorescein, and ruling out the presence of excess fluorescein by IR. Dendrimer properties are: first generation, soluble in MeOH/EtOH/H_2_O, and molecular weight of 2234.66 g/Mol.

The final product in our conditions showed a high stability in all experiments.

#### Structural Characterization

G_1_-[(NCPh(o-N))_3_NH_2_] (**1**), ^1^H-NMR (CD_3_OD): δ (ppm) 0.02 (s, 24H, -(CH_3_)_2_Si-); 0.59 (m, 24H, -SiCH_2_-); 1.38 (m, 8H, -SiCH_2_CH_2_CH_2_Si); 1.46 (m, 2H, -SiCH_2_CH_2_CH_2_NH_2_); 1.73 (m, 6H, -SiCH_2_CH_2_CH_2_N); 2.61 (t, 2H, -CH_2_NH_2_); 3.66 (m, 6H, -CH_2_N); 7.46 (m, 3H, NCHCH^Ar^); 7.89 (t, 3H, CCHCH^Ar^); 8.00 (d, 3H, CCH^Ar^); 8.35 (s, 3H, -CH=N); 8.61 (m, 3H, NCH^Ar^). Elemental Analysis (%): Calc. C_50_H_89_N_7_Si_5_ (928.72): C, 64.66; H, 9.66; N, 10.56; Found: C, 64.30; H, 9.00; N, 10.48.


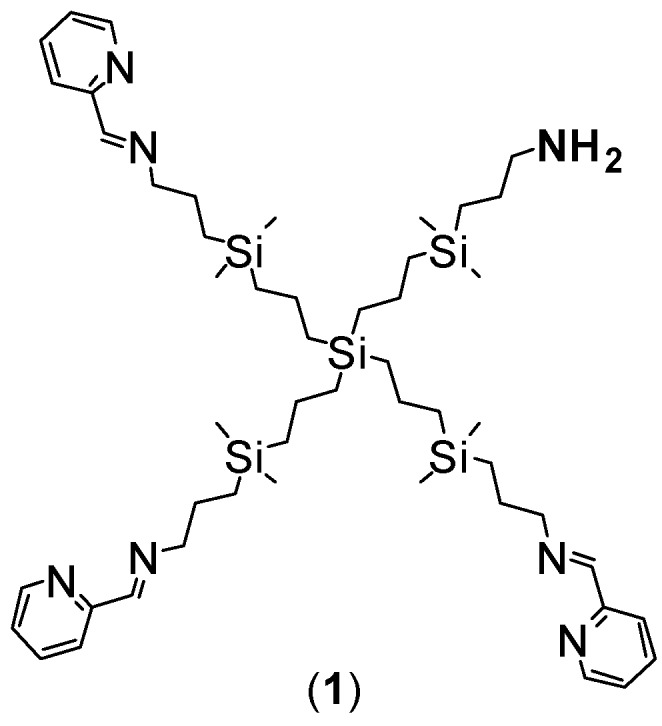


G_1_-[(NCPh(o-N)Ru(η^6^-p-cymene)Cl_2_)_3_NH_2_] (**2**),^1^H-NMR (CD_3_OD): δ (ppm) 0.02 (s, 24H, -(CH_3_)_2_Si-); 0.61 (m, 24H, -SiCH_2_-); 1.02&1.15 (2d, 18H, CH(CH_3_)_2_^cym^); 1.38 (m, 8H, -SiCH_2_CH_2_CH_2_Si); 1.82 (m, 2H, -SiCH_2_CH_2_CH_2_NH_2_); 1.97 (m, 6H, -SiCH_2_CH_2_CH_2_N); 2.27 (s, 9H, CCH_3_^cym^); 2.67 (m, 3H, CH(CH_3_)_2_^cym^); 2.86 (t, 2H, -CH_2_NH_2_); 4.25&4.68 (2m, 6H, -CH_2_N); 5.81 (m, 6H, CHCCH_3_^cym^); 6.10 (dd, 6H, CHCCH(CH_3_)_2_^cym^); 7.76 (m, 3H, NCHCH^Ar^); 8.13 (m, 3H, CCHCH^Ar^); 8.20 (m, 3H, CCH^Ar^); 8.65 (s, 3H, -CH=N); 9.47 (m, 3H, NCH^Ar^). Elemental Analysis (%): Calc. For C_80_H_131_Cl_6_N_7_Ru_3_Si_5_ (1847.30): C, 52.01; H, 7.15; N, 5.31; Found: C, 51.74; H, 7.08; N, 5.30.


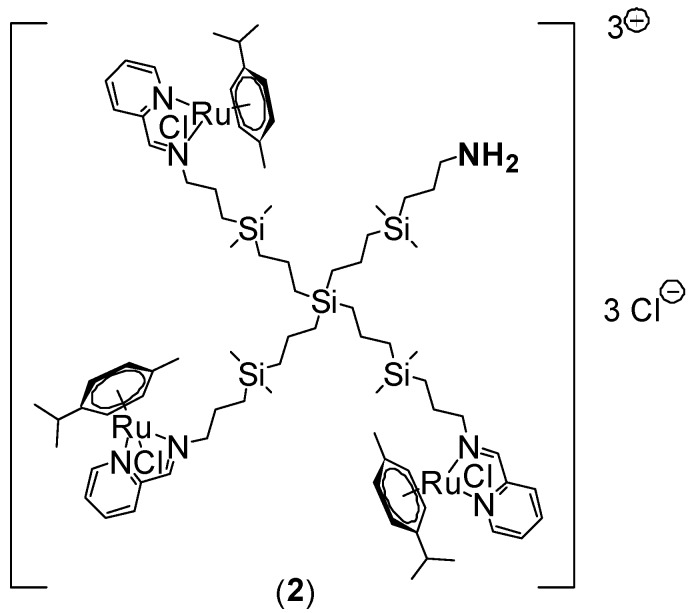


G_1_[(NCPh(o-N)Ru(η^6^-p-cymene)Cl_2_)_3_NH_2_-FITC] (**3**)**,** ^1^H-NMR (CD_3_OD): δ (ppm) 0.02 (s, 24H, -(CH_3_)_2_Si-); 0.61 (m, 24H, -SiCH_2_-); 1.02&1.15 (2d, 18H, CH(CH_3_)_2_^cym^); 1.38 (m, 8H, -SiCH_2_CH_2_CH_2_Si); 1.82 (m, 2H, -SiCH_2_CH_2_CH_2_NH_2_); 1.97 (m, 6H, -SiCH_2_CH_2_CH_2_N); 2.27 (s, 9H, CCH_3_^cym^); 2.67 (m, 3H, CH(CH_3_)_2_^cym^); 3.30 (t, 2H, -CH_2_NHSNH); 4.25&4.68 (2m, 6H, -CH_2_N); 5.81 (m, 6H, CHCCH_3_^cym^); 6.10 (dd, 6H, CHCCH(CH_3_)_2_^cym^); 6.60 (m, FITC); 7.76 (m, 3H, NCHCH^Ar^); 8.13 (m, 3H, CCHCH^Ar^); 8.20 (m, 3H, CCH^Ar^); 8.65 (s, 3H, -CH=N); 9.47 (m, 3H, NCH^Ar^). UV-Vis: 507 nm (FITC). Elemental Analysis (%): Calc. For C_101_H_140_Cl_6_N_8_O_5_Ru_3_SSi_5_ (2234.66): C, 54.24; H, 6.40; N, 5.01; S, 1.43 Found: C, 54.19; H, 6.29; N, 5.02; S, 0.99.


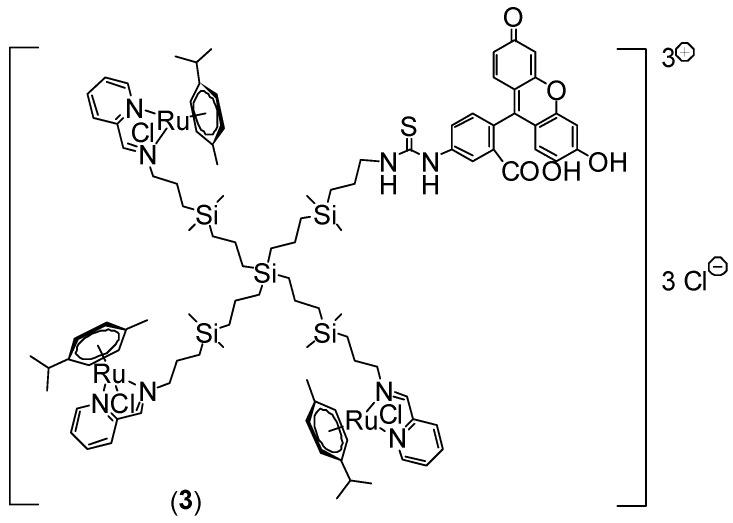


### 3.2. Zeta Potential and Particle Size

Zeta potential and zeta size of CRD13-FITC dendrimer were 25.8 ± 3.2 mV and 476 ± 54 nm, respectively.

### 3.3. Transmission Electron Microscopy

**Figure 4:**
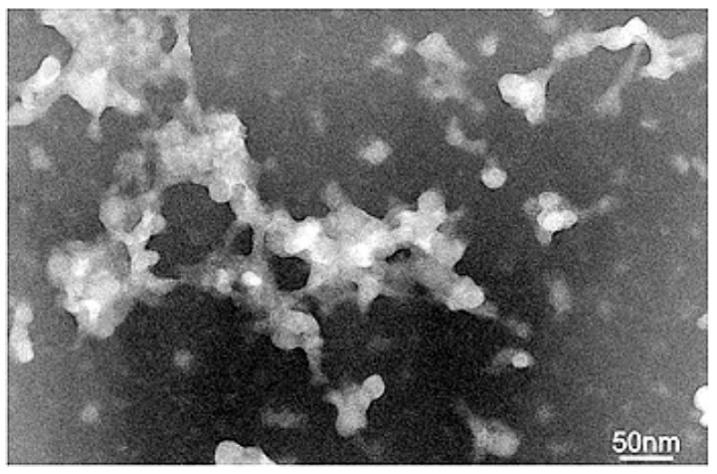
Ultrastructure of ruthenium terminated carbosilane dendrimer with FITC (**CRD13-FITC**). Dendrimer was dissolved in Na-phosphate buffer at 10 mmol/L, and pH7.4. Bar = 50 nm.

The CRD13-FITC dendrimer produced characteristic branched structures but some single nanoparticles were also observed. The size of aggregates and single nanoparticles was from 50 to 500 nm and from 5 to 10 nm, respectively (Figure 4).

### 3.4. Haemotoxicity

**Figure 5:**
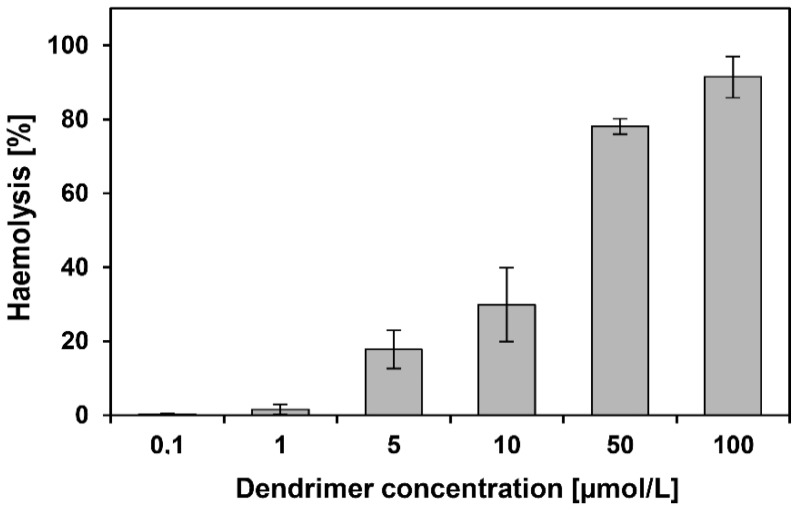
Haemolysis induced after 24h treatment of red blood cells with ruthenium terminated carbosilane dendrimer with FITC (CRD13-FITC) at 0.1–100 µmol/L. Two percent haematocrit in PBS buffer at pH 7.4 and 22 °C. Results are mean ± SD, *n* = 6.

Haemotoxicity is a simple method to investigate the disruption of cell membrane caused by chemical compounds. CRD13-FITC dendrimer haemolysed erythrocytes at 50 and 100 µmol/L, but, at lower concentrations of 0.5–10 µmol/L, the haemolysis did not exceed 30% of control (Figure 5). The haemolysis of erythrocytes in water was chosen as a positive control and it was added in the formula to calculate the haemolysis.

### 3.5. Fluorescence Anisotropy

**Figure 6:**
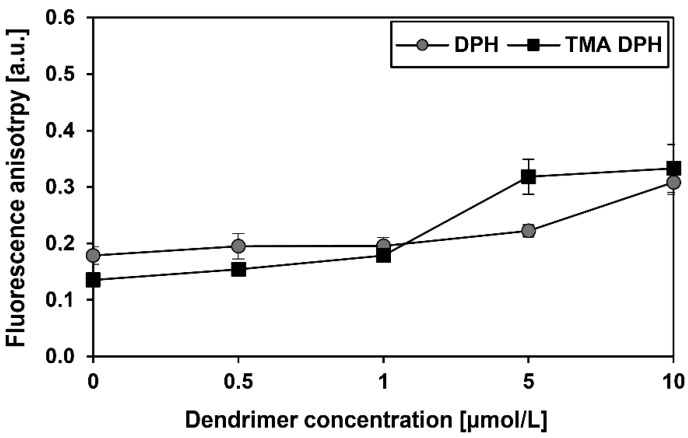
Changes in fluorescence anisotropy of DPH and TMA-DPH of erythrocyte membranes treated with ruthenium terminated carbosilane dendrimer with FITC (CRD13-FITC) at rising concentrations from 0.5 to 10 µmol/L, PBS buffer, pH 7.4, 37 °C. The values are the mean ± SD n = 3.

To explain the interaction of CRD13-FITC dendrimer with red blood cell membranes, anisotropy changes were investigated. Increased fluorescence anisotropy of TMA-DPH is responsible for the increase of the rigidity of hydrophilic regions of the membrane. In turn, the increase in DPH anisotropy corresponds to a decrease in the fluidity of the hydrophobic phospholipid bilayer. The presented results (Figure 6) indicate that CRD13-FITC at concentrations of 0.5–10 µmol/L changed anisotropy of samples. Increased fluorescence anisotropy of both probes was dose-dependent indicative of changes in membrane fluidity. Increase of DPH and TMA-DPH fluorescence anisotropy in the presence of CRD13-FITC dendrimer indicated that it interacts with both hydrophobic and hydrophilic regions of the membrane.

### 3.6. Viability

**Figure 7:**
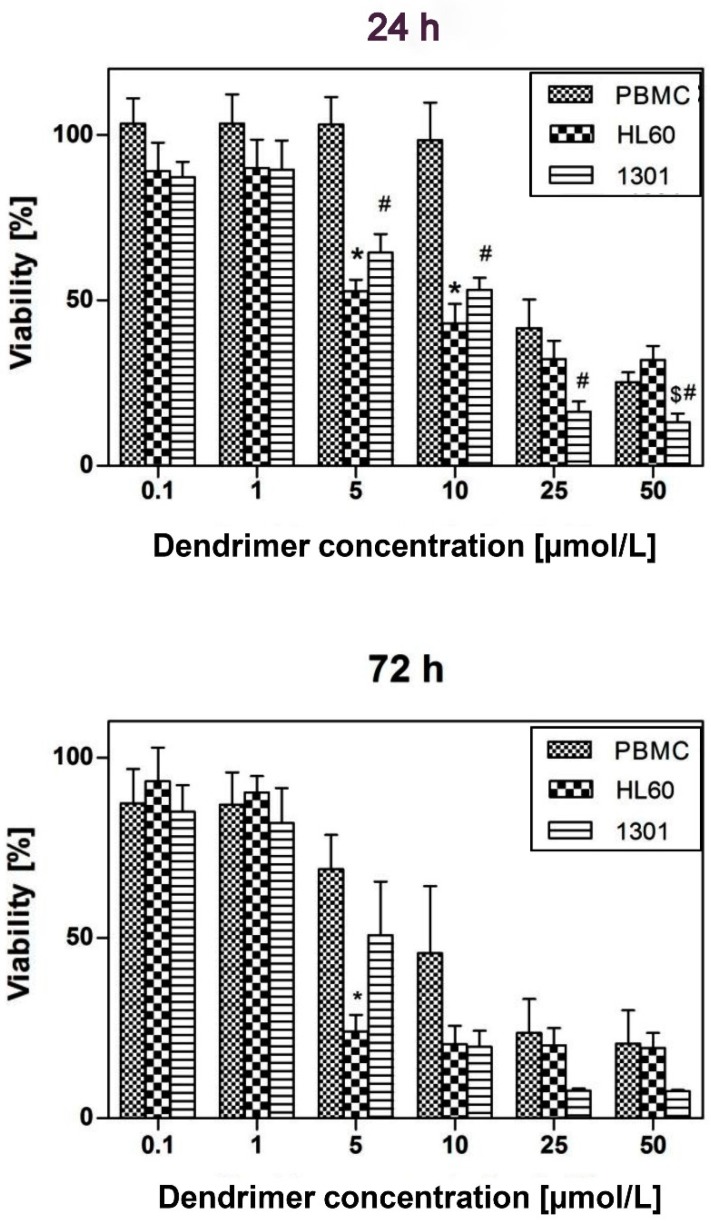
Effect of ruthenium terminated carbosilane dendrimer with FITC (CRD13-FITC) on the viability of PBMC, HL-60 and 1301 cells after 24 h and 72 h incubation. The values are the mean ± SD, *n* = 9. Statistically significant differences in comparison to the control cells (* *p* <0.05, # *p* <0.01, ^$^ *p* <0.001).

We estimated the effects of CRD13-FITC dendrimer on the viability of PBMC normal cells as well as HL60 and 1301 leukemic cells after 24 and 72 h incubation (Figure 7). The viability of cells upon treatment with DMSO (100% cell death) was chosen as a positive control and it was added into the formula to calculate viability. Cell viability decrease was time- and dose-dependent. The dendrimers up to 10 µmol/L, after 24 and 72 h of incubation, were less cytotoxic for normal cells than for both cancer cell lines. The viability of PBMC cells in the presence of dendrimer at up to 5 µmol/L was slightly reduced. In contrast, the viability of cancer HL-60 and 1301 cells was significantly decreased in the presence of CRD 13-FITC. The IC_50_ values after 72 h incubation were lower (8.04 ± 1.01 for PBMC cells, 2.26 ± 1.01 for HL60, 5.45 ± 1.01 for 1301 cell lines, *p* < 0.05 between normal and cancer cells, *p* < 0.05 between HL60 and 1301) in comparison to 24 h incubation (18.9 ± 1.0 for PBMC, 3.74 ± 1.09 for HL60 and 10.32 ± 1.15 for 1301 cells, *p* < 0.05 between normal and cancer cells, *p* < 0.05 between HL60 and 1301).

### 3.7. Cellular Uptake

**Figure 8:**
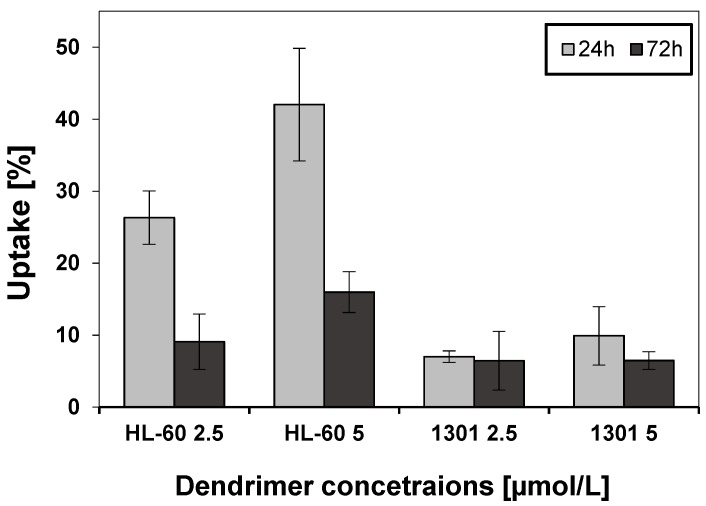
Flow cytometry of the uptake by HL-60 and 1301 cells of ruthenium terminated carbosilane dendrimer with FITC (CRD13-FITC). The results are mean ± SD, *n* = 3.

**Figure 9:**
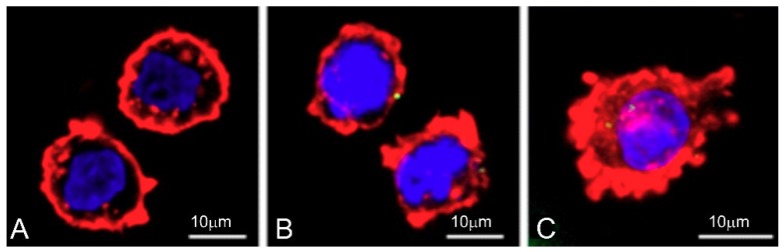
Confocal microscopy images of HL-60 cells after 24 h incubation with ruthenium terminated carbosilane dendrimer with FITC (CRD13-FITC). A-control, B-2.5 µmol/L and C-5 µmol/L. Bar = 10 µm.

Cellular uptake of CRD13 dendrimer labelled with fluorescein (CRD13 FITC) at 2.5 and 5 µmol/L by HL-60 and 1301 cell lines was monitored after 24 and 72 h (Figure 8 and Figure 9). Cytometer and confocal investigations showed that the dendrimer was taken up more intensively by HL-60 cells than 1301 cells. The percentage of cellular uptake for HL-60 cells was higher at both concentrations after 24 h than after 72 h. In both cell lines, internalisation of CRD-FITC dendrimer was higher at 5 than 2.5 µmol/L. As a positive control, the cellular uptake of corresponding non-modified carbosilane ruthenium dendrimer was chosen that was equal to 30 ± 3% at 24 h in HL60 cells.

## 4. Discussion

The investigated dendrimer has a positive zeta potential of 25.8 ± 3.2mV. Similar results were demonstrated for CRD13 (without modification with fluorescein), i.e., 21.5 ± 6.2mV. The higher-generation dendrimer has a correspondingly higher zeta potential [5]. Positive charges of both dendrimers indicate that they should readily interact with negatively charged cell membrane [5,23,24]. Due to interaction between dendrimers, they can form aggregates. In aqueous solution, the size of the CRD13-FITC nanoparticle was 476.8 ± 54.4 nm, but this dendrimer created characteristic 50–500 nm branched structures in the dry state. Sometimes 4–10 nm single nanoparticles were observed. Differences between the results obtained in a solution and in a dry state are well known [5,25,26]. The zeta size of the unmodified CRD13 dendrimer was 455.7 ± 139.0 nm (as was CRD13 dendrimer), whereas 50–800 nm aggregates were visible on the TEM images [5]. Their respective results appear very similar, which indicates that addition of fluorescein changes neither the size nor the potential of the dendrimer. Our dendrimer caused haemolysis after 24 h incubation mainly at higher concentrations. Similarly, CRD13 disrupted membranes at concentrations from 25 to 100 µmol/L after 24 h incubation [5]. The fact that haemolytic activity depends on dendrimer concentration has already been shown earlier for carbosilane [27,28], phosphorus [29] and PAMAM dendrimers [30,31]. Due to surface charge, cationic dendrimers can react with negatively charged and damage red blood cell membranes more than neutral or anionic ones [32,33,34]. Therefore, on the one hand, positive surface charge increases the chance that a nanoparticle will cross the membrane barrier, while, on the other hand, it can lead to membrane damage and cell death [35]. CRD13-FITC at concentrations of 0.5–10 µmol/L interacting with both hydrophobic and hydrophilic regions of the membrane changes their fluidity. A similar effect was observed for the unmodified CRD13 dendrimer (unpublished data). Interactions of other carbosilane dendrimers with hydrophobic and hydrophilic regions of the membrane have also previously been demonstrated [36]. The PBMC, HL-60 and 1301 cell viability after incubation with CRD13-FITC dendrimer decreased with time. Cytotoxicity to normal PBMC cells was lower than with both cancer cell lines. The viability of PBMC cells in the presence of CRD13-FITC dendrimer at up to 5 µmol/L was slightly reduced after 24 and 72 h treatment. In contrast, the viability of HL-60 and 1301 cancer cells was significantly decreased in the presence of CRD 13-FITC at the same concentration. This is in agreement with the previous work for PBMC (unpublished data), HL-60 and 1301 cells treated with unlabelled CRD13 dendrimer [5]. A similar effect of carbosilane dendrimers on other cancer cell types was reported by Maroto-Diaz et al. [37]. Absence of a negative influence of carbosilane dendrimers on the normal cells was also noted [27,38]. In contrast, significant effects of ruthenium containing complexes were reported [39,40]. Ruthenium is a metal with anti-tumour properties [39,41]. Our dendrimer was more toxic for cancer than normal cells, probably due to the presence of ruthenium. IC_50_ values after 72 h incubation with CRD13-FITC dendrimer were lower in comparison to after 24 h, giving results in agreement with others in the literature [5]. Flow cytometry and confocal microscopy techniques were used to assess the uptake efficiency of CRD13 FITC. The dendrimer at 5 µmol/L was taken up by HL-60 and 1301 cell lines, and was more effective than at 2.5 µmol/L. Both techniques showed that CRD13-FITC dendrimer was more intensively internalized by HL-60 than 1301 cells. Moreover, the uptake of the dendrimer was more intensive after 24 than after 72 h in HL-60 cells, probably due to its higher toxicity after longer incubation. PAMAM FITC labelled dendrimers can enter C6 cells after 12 h [42]. A similar effect was observed for complexes of CRD13 dendrimer and fluorescently labelled siRNA taken up by HL-60 cells after 3 h [43]. Other dendritic molecular particles have been described as good transporters of bioactive cargos into cells [44].

## 5. Conclusions

Thus, the labelling of carbosilane ruthenium dendrimer by FITC did not decrease its uptake into cells in comparison with the non-labelled one. On the other hand, the addition of a fluorescent probe allowed tracking the metallodendrimer in normal and cancer cells. It was found that carbosilane ruthenium dendrimer labelled with FITC in concentration up to 10 µmol/L was more cytotoxic for cancer cells than for normal cells. These data are in good agreement with our data with non-labelled carbosilane ruthenium dendrimer. FITC-labelled carbosilane ruthenium dendrimer was internalized more intensively into HL-60 (suspension culture) than into 1301 adhesive cells. Thus, FITC labelled carbosilane ruthenium dendrimer is a good candidate for diagnostic imaging and studying anticancer effects of metallodendrimers in cancer therapy.

## Supplementary Materials

The following are available online at https://www.mdpi.com/2218-273X/9/9/411/s1, Figure S1: ^1^H-NMR spectrum of compound **1** in CD_3_OD, Figure S2. ^1^H-NMR spectrum of compound **2** in CD_3_OD, Figure S3. DOSY spectrum of compound **3** in CD_3_OD.
