# Supplementary material for: Synthesis and Characterization of FITC Labelled Ruthenium Dendrimer as a Prospective Anticancer Drug
Source: Biomolecules. 2019 Aug 25;9(9):411. doi: 10.3390/biom9090411 (PMC6770823; doi:10.3390/biom9090411)
Supplement: Supplementary file 1 [file biomolecules-09-00411-s001.pdf]

SUPPORTING INFORMATION

Synthesis and characterization of FITC labelled ruthenium dendrimer a controlled carrier in anticancer drug delivery

General Information

Elemental Analysis. C, H and N elemental analysis was performed in a microanalyzer LECO CHNS-932.
Nuclear Magnetic Resonance (NMR). ^{1}H-NMR experiments were performed on Varian Unity-500, Unity-300 and Mercury-300 instruments. $CDCl_3$ and CD_3OD were used as solvents. TOCSY and DOSY experiments were performed on selected compounds to confirm characterization details.
UV-Vis spectrophotometry. UV-Vis spectra were recorded using a standard PerkinElmer Lambda 35 spectrophotometer, in the range λ = 200–900 nm and using water as solvent.
FT-IR spectroscopy. FT-IR spectra were obtained using a PerkinElmer Frontier spectrometer, over KBr solid samples in the range 4000–400 cm^{-1}.

Figures

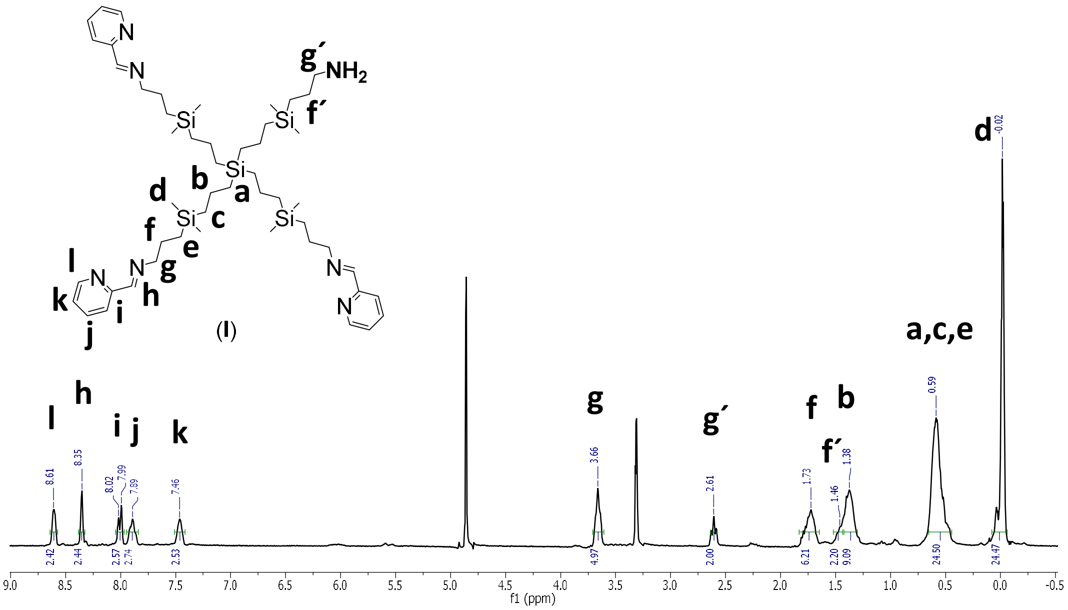

Figure S1. ^{1}H-NMR spectrum of compound **1** in CD_3OD.

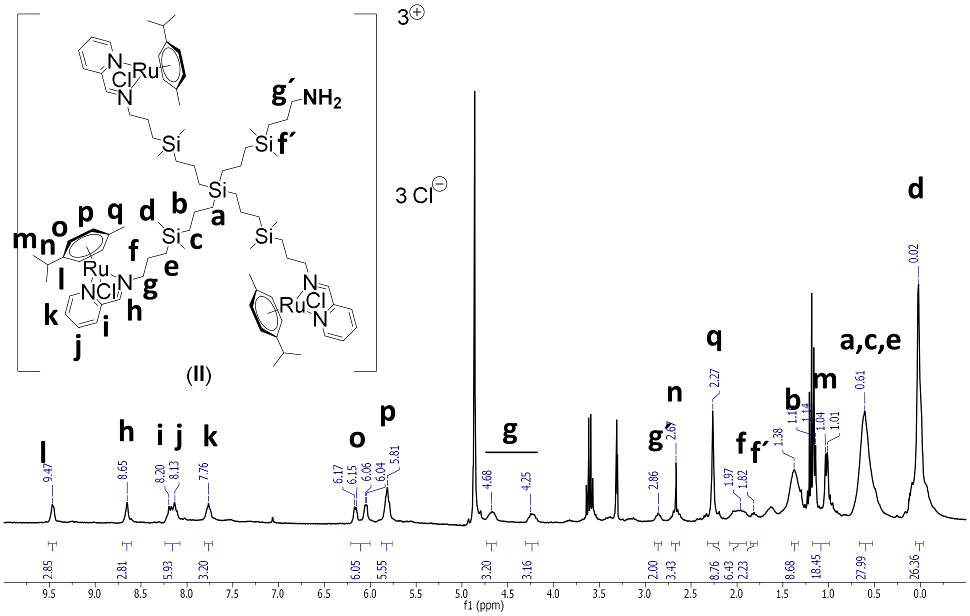

Figure S2. ^{1}H-NMR spectrum of compound **2** in CD_3OD.

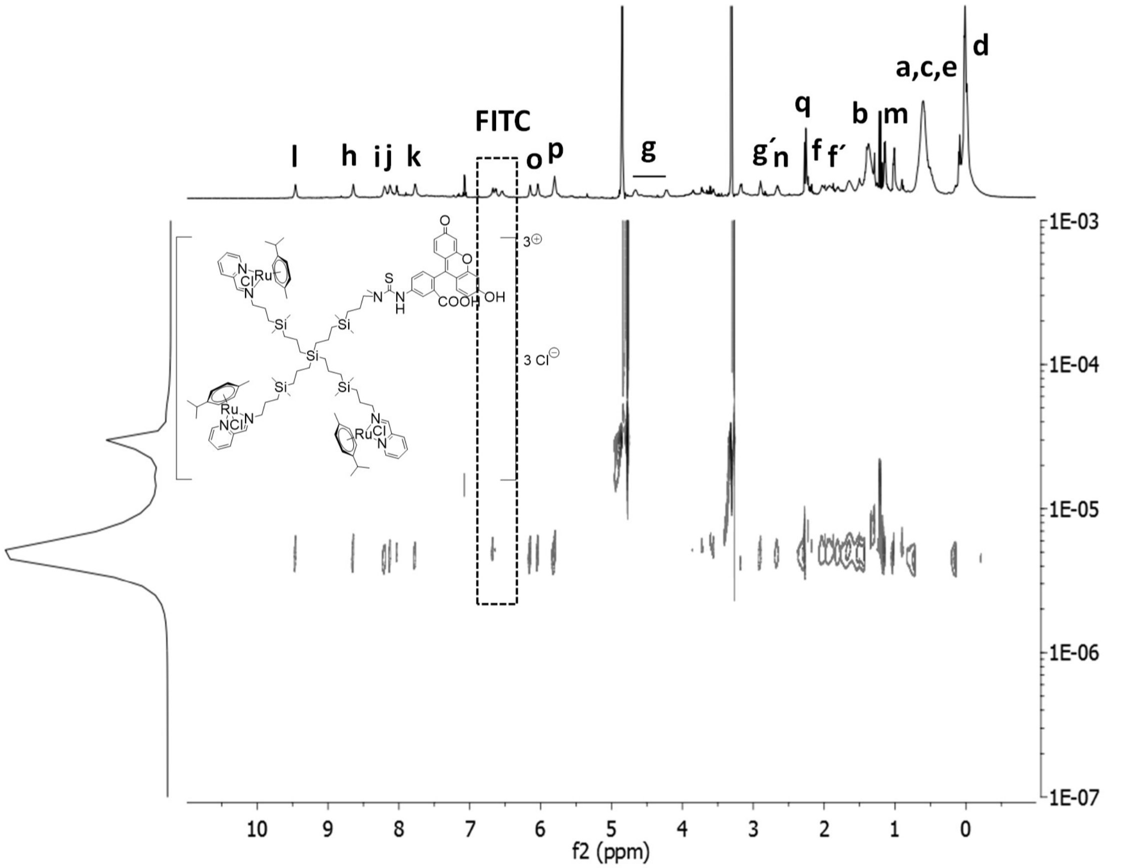

Figure S3. DOSY spectrum of compound **3** in CD_3OD
